# Standardization and normative data of the 48-item Yoni short version for the assessment of theory of mind in typical and atypical conditions

**DOI:** 10.3389/fnagi.2022.1048599

**Published:** 2023-01-12

**Authors:** Sara Isernia, Federica Rossetto, Simone Shamay-Tsoory, Antonella Marchetti, Francesca Baglio

**Affiliations:** ^1^IRCCS Fondazione Don Carlo Gnocchi ONLUS, Milan, Italy; ^2^Department of Psychology, University of Haifa, Haifa, Israel; ^3^Research Unit on Theory of Mind, Department of Psychology, Università Cattolica del Sacro Cuore, Milan, Italy

**Keywords:** social cognition, validation, theory of mind, neurocognitive assessment, mentalizing, normative data, rehabilitation

## Abstract

**Introduction:**

The Yoni task is a computerized tool assessing first-and second-order affective and cognitive Theory of Mind (ToM), accounting for the multidimensional and multi-level mentalizing features. The Italian Yoni task has been validated and standardized in its 98-item version, and a 48-item short version has been proposed for a quick digital evaluation of ToM in clinical contexts.

**Methods:**

The present study aimed to test the Yoni-48’s convergent validity, correlating the tool score with the Reading the Mind in the Eyes test (ET) and Gender Test (GT), its items discrimination ability through the Classical Test Theory, and Rash model, its reliability by evaluating the internal consistency (McDonald’s *ω*, Cronbach’s *α*, Guttman’s *λ*^2^, and Guttman’s *λ*^6^) and Spearman-Brown ϱ_SP_ split-half analysis, and to provide standardization and normative data in the Italian population.

**Results:**

Results suggested a good convergent validity with a statistically significant association with ET (*p* < 0.001), while a null correlation was observed with GT (*p* = 0.947). The Classical Test Theory and Rash model confirmed a good discrimination ability of the Yoni-48’s second-order affective and cognitive ToM items, while weaker discrimination capacity was registered for the first-order ToM items. The inter-item reliability was optimal for clinical purposes (*ω*, *α*, *λ*^2^, *λ*^6^ ≥ 0.90). Also, the split-half reliability was high (Spearman-Brown ϱ_SP_ = 0.90). For standardization, age and education were revealed as significant predictors of Yoni accuracy scores, except for the first-order ToM score. Instead, age was the only predictor of Yoni’s response speed score. The Italian normative data showed a high Yoni accuracy in healthy adults (mean accuracy = 0.85) and speed (mean response time = 0.92). Finally, both accuracy and response time level was balanced between the affective and cognitive components of ToM.

**Discussion:**

This study supports the psychometric properties of the Yoni-48 and provides normative data for the Italian population. Further studies are needed to test the suitability of this short version for profiling the social cognition neurocognitive phenotype.

## 1. Introduction

Theory of Mind (ToM) refers to mental processes allowing individuals to understand other’s mental states, which can be different from one’s own, driving behaviors and actions. ToM has been defined as a complex multi-component psychological construct that includes the ability to make inferences about beliefs and thoughts (cognitive ToM) and desires and emotions (affective ToM; Shamay-Tsoory and Aharon-Peretz, 2007; Kalbe et al., 2010; Sebastian et al., 2012; Baglio and Marchetti, 2016), with a hierarchical level of mental states attribution. Developmental research identified key stages of ToM evolution, which implies incremental complexity of meta-representational attributions. The first-order inferences (e.g., “*he/she thinks that…*”), which leads individuals to represent the mental states of others and compare them with their own, constitutes the first developmental milestone in the understanding of other’s mental states, and usually acquired around 4–5 years of age (Wimmer and Perner, 1983; Perner and Wimmer, 1985; Shamay-Tsoory et al., 2005; Kalbe et al., 2010; Ruitenberg et al., 2020). Then, the second level of recursive thinking (e.g., “*he/she thinks that he/she thinks that…*”), named the second-order ToM inference, emerges around 8 years of age, and requires an individual to represent two other individuals’ mental states (Wimmer and Perner, 1983; Perner and Wimmer, 1985; Shamay-Tsoory et al., 2005; Kalbe et al., 2010; Ruitenberg et al., 2020). With advancing age, an inverse trend is observed, with an age-related decline starting from the complex ToM tasks such as the second-order ToM tasks (Maylor et al., 2002; Charlton et al., 2009; Phillips et al., 2011; Cavallini et al., 2013). Multiple studies supported ToM level as a function of age (Duval et al., 2011; Cavallini et al., 2013; Henry et al., 2013; Moran, 2013; Bottiroli et al., 2016; Rosi et al., 2016; Klindt et al., 2017). However, this association seems to be mediated and moderated by education level and cognitive functions (Phillips et al., 2011; Rakoczy et al., 2012; Li et al., 2013), especially executive functions (Charlton et al., 2009; Ibanez et al., 2013; Bottiroli et al., 2016). In fact, age-related changes in executive functions may explain the different ToM levels in older adults and clinical conditions (Wade et al., 2018; Chainay and Gaubert, 2020; Otsuka et al., 2021). This is also in accord with a recent ToM neural model supporting core mentalizing circuits relying on frontal loop networks (Abu-Akel and Shamay-Tsoory, 2011). Recent works especially support the essential role of inhibitory control and cognitive flexibility on mental states understanding, fueling the debate about the relationship between executive functions and ToM (Roca, 2016). The evidence-based findings on this issue currently remain inconclusive and may be related to the fact that ToM subdimensions are often neglected and not considered in these studies (Roca, 2016).

The selective impairment of affective and cognitive ToM is now considered a hallmark of several neuro-psychiatric conditions (Henry et al., 2016), and the Diagnostic and Statistical Manual of Mental Disorders, fifth edition (American Psychiatric Association, 2013), reported socio-cognitive functioning as one of the six cores of neurocognitive functions, relevant to the diagnosis. However, different from the other neurocognitive functions, standardized social cognition tools are still a few. Moreover, digital social cognition tools are needed to avoid labor methods for data collection, automatizing, and standardizing the administration modality in accordance with the recent migration of neuropsychology toward the digitalization of tests (Weintraub et al., 2018).

The Yoni task (Shamay-Tsoory and Aharon-Peretz, 2007; Rossetto et al., 2018; Isernia et al., 2022) is a computerized test for the assessment of various mentalizing components and levels, accounting for the multidimensional and multilevel features of ToM. The Yoni task has been originally used in the research field to investigate social cognitive deterioration in many atypical conditions, especially the ones hallmarked by a dissociated impairment in different ToM components. For example, different performances in affective and cognitive ToM were reported in patients with localized brain lesions (Abu-Akel and Shamay-Tsoory, 2011), schizophrenia (Shamay-Tsoory et al., 2010), Parkinson’s Disease (Bodden et al., 2010; Rossetto et al., 2018), and Mild Cognitive Impairment (Rossetto et al., 2018). Our recent work (Isernia et al., 2022) revealed the Yoni-98 as a valid and reliable ToM measure with adequate internal consistency also for clinical purposes. Normative data and two composite scores were obtained in the Italian population: the global level of ToM and the balance between affective and cognitive ToM, both in terms of accuracy and response time. Moreover, given the need to provide tools agile to be adopted for first-level neuropsychological batteries, two short versions (the 48-items version, Yoni-48, and the 36-items version, Yoni-36) have been developed from the 98-items version, with a balanced number of items evaluating each subdomain of mentalizing. However, the psychometric properties, standardization, and normative data of these short-versions still need to be reported.

The present study aimed to test (1) the Yoni-48 validity (convergent and discriminant validity, and items discrimination ability; Isernia et al., 2022); (2) inter-item reliability (internal consistency and split-half reliability); and to (3) standardize and provide normative data in the Italian population.

## 2. Materials and methods

A primary prospective cross-sectional study was conducted after approval of the IRCCS Don Gnocchi Foundation Ethics Committee. Data were collected from January to August 2022 in line with the Declaration of Helsinki. Participants read and signed the written informed consent module before taking part in the research study.

### 2.1. Participants

Subjects were consecutively enrolled at the IRCCS Don Gnocchi Foundation in Milan (staff, volunteers, students, and patients’ caregivers) and recruited as eligible for the research according to the following inclusion/exclusion criteria: age >18; years of education ≥5; absence of a history of neurological, psychiatric, and/or relevant organic conditions as investigated during a clinical interview; absence of auditory and visual disability plausibly affecting the performance of test battery; absence of pharmacological treatment plausibly affecting the test battery performance.

All the subjects voluntarily took part in the study and did not receive compensation for their participation in the research.

### 2.2. Materials

Participants were administered the computerized version of the Italian Yoni short version (Yoni-48; Isernia et al., 2022) and the Reading the Mind in the Eyes Test (ET; Baron-Cohen et al., 2001) in a single individual session lasting about 20 min.

#### 2.2.1. Yoni-48

The Yoni-48 is the short version of the Italian Yoni task (Rossetto et al., 2018) for the assessment of ToM, including 48 items in total: 42 mental and 6 physical stimuli. The mental stimuli comprise 21 affective and 21 cognitive ToM items and are divided into 16 first-order (8 affective, 8 cognitive) and 26 second-order (13 affective and 13 cognitive) ToM stimuli. The task shows visual cartoon-like stimuli, in which a face named “Yoni” appeared at the center of the screen, surrounded by four colored pictures (fruit, animals, means of transport, or faces). Subjects are instructed to click as fast as they can on the picture to which Yoni refers, based on the sentence reported at the top of the screen (e.g., *“Yoni is thinking of …,” “Yoni likes …”*), the Yoni’s eyes gaze and facial expression, and the eyes gaze and facial expression of faces around Yoni. Sixty seconds are available at maximum to answer each item. For each item, only one answer is correct (score 0–1). Also, the response time (seconds) for each item is recorded (score 0–60). Yoni total raw score, first-order raw score, second-order raw score, affective raw score, and cognitive raw score are computed by summing items score for accuracy and by averaging items scores for response time (instructions for scoring are reported in Supplementary materia1 S1). Then, two composite scores are obtained for accuracy and response time, referring to the performance level and the balance between cognitive and affective ToM performance: the accuracy composite score (ACC), the response time composite score (RT; range 0–1), the Cognitive/Affective accuracy index (CA_A_), and the Cognitive/Affective response time index (CA_RT_) (Details on the procedure to compute composite scores are reported in Statistical Analysis).

#### 2.2.2. Reading the mind in the eyes test

The ET is a validated measure of advanced ToM (Vellante et al., 2013; Preti et al., 2017; Black and An, 2019; Maddaluno et al., 2022). Thirty-six black-and-white photographs of males’ and females’ gazes expressing complex mental states are shown with four verbal alternatives. The subject is instructed to choose the mental state fitting the gaze of the photograph. Each item is scored 0–1, for a total score ranging from 0 to 36. The same stimuli are also administered with different instructions, indicating whether the photo depicts a male or female, as a control task (Gender Task, GT).

### 2.3. Statistical analysis

JASP (version 0.16.1.0; JASP Team 2020) and IBM SPSS Statistics (version 28.0.1.1) were used for the statistical analysis.

*Participants’ demographics:* Frequency, mean, median, and standard deviation were computed to describe the demographical characteristics of the participants. Chi-Squared was run to test the distribution differences between males and females in each age-and education-group.

*Yoni-48 validity:* Spearman’s ϱ correlation was performed to test the association between the Yoni-48 total score and its subscales and the ET (convergent validity) and between the Yoni-48 total score and its subscales and the GT (discriminant validity). An alpha threshold of 0.005 was considered for Bonferroni’s multiple comparison correction. The Yoni-48 item discrimination ability was computed by dividing the sample into two sub-groups based on the median of the Yoni-48 total score (high and low score group) and extracting the effect size *h* of the Ebel index *D*. The item discrimination ability was also tested with the dichotomous Rash model (Item Response Theory), by computing information-weighted mean square statistic and outlier sensitive means square statistic. Pearson’s reliability coefficient was reported to test the model’s accuracy.

*Yoni-48 reliability:* Inter-item reliability was tested through McDonald’s ω, Cronbach’s α, Guttman’s *λ*^2^, and Guttman’s *λ*^6^. Also, split-half was run with parallel reliability Spearman-Brown ϱ_SP_.

*Yoni-48 standardization:* To test the effect of sex, age, and education years on Yoni-48 components and levels, simultaneous multivariate regression models were performed. *β*-values and means of the predictor variables were inserted in a formula to adjust raw scores of Yoni-48 accuracy and response time. The formula was computed for each Yoni-48 component and level to extract the adjustment score table.

*Yoni-48 composite scores:* Yoni-48 raw scores (Yoni affective, cognitive, first-level, second-level ToM score) were adjusted for sex, age, and education years according to the adjustment formula. The upper and inner scores of Yoni-48 accuracy and response time were not adjusted. Then, ACC, RT, CA_A_, and CA_RT_ composite scores were computed.

To calculate the ACC, the first-order score (0–16) and the second-order (0–26) total raw score were computed by separately summing the score of the sub-scales items. Then, first-and second-order total raw scores were adjusted for sex, age, and education. Finally, the composite accuracy score was computed by summing the first-and second-order adjusted scores and dividing the result by the number of mental items for a total score ranging from 0 to 1. An ACC score near 1 indicates a high level of performance.


$$ACC=\left(\begin{array}{l} \mathrm{first-order adjusted}\;\mathrm{ToM}\;\mathrm{accuracy}+\\ \mathrm{second-order adjusted}\;\mathrm{ToM}\;\mathrm{accuracy} \end{array}\right)/N_{i}$$

To calculate the RT, the mean of the first-and second-order items’ response time(s) was separately computed (score 0–60). Then, both scores were adjusted for sex, age, and education. Finally, the RT was obtained by subtracting 1 to the average of the sum of the first-order adjusted ToM RT(s) minus the minimum response time(s) available per item on the total available time per item and the second-order adjusted ToM response time(s) minus the minimum response time(s) available per item on the total available time per item. According to the ACC, the RT score ranges from 0 to 1. An RT score near 1 indicates a high level of performance (fast response).


$$RT=1-\left(\left(\begin{array}{l} \left(\left(\mathrm{first-order adjusted}\;\mathrm{ToM}\;\mathrm{RT}-{\mathrm{RT}}_{\min}\right)/{\mathrm{RT}}_{i}\right)\\ +\left(\left(\mathrm{second-order adjusted}\;\mathrm{ToM}\;\mathrm{RT}-{\mathrm{RT}}_{\min}\right)/{\mathrm{RT}}_{i}\right) \end{array}\right)\right)/2$$

The CA_A_ and CA_RT_ score was computed to detect an eventual dissociation between the level of cognitive and affective ToM performance.

To compute the CA_A_, the affective and cognitive ToM accuracy raw total scores were computed separately (score 0–21) by summing the score of the items of the subscales. Then both scores were adjusted for sex, age, and education. Finally, the CA_A_ score was obtained with the formula as follows:


$$CA_{A}=\;\;\begin{array}{c} \;\left(\begin{array}{l} \mathrm{affective adjusted}\;\mathrm{ToM}\;\mathrm{accuracy}\\ -\mathrm{cognitive adjusted}\;\mathrm{ToM}\;\mathrm{accuracy} \end{array}\right)\\ \;\;/\;\left(\begin{array}{l} \mathrm{affective adjusted}\;\mathrm{ToM}\;\mathrm{accuracy}\\ +\mathrm{cognitive adjusted}\;\mathrm{ToM}\;\mathrm{accuracy} \end{array}\right) \end{array}\;$$

To compute the CA_RT_, the affective and cognitive RT raw total scores were computed separately (score 0–60) by averaging the score of each item. Then both two scores were adjusted for sex, age, and education. Finally, the CA_RT_ score was obtained with the formula as follows:


$$CA_{RT}=\left(\begin{array}{l} \mathrm{affective adjusted}\;\mathrm{ToM}\;\mathrm{RT}-\\ \mathrm{cognitive adjusted}\;\mathrm{ToM}\;\mathrm{RT} \end{array}\right)*-1$$

A CA_A_/CA_RT_ score near 0 suggests a balance between cognitive and affective ToM performance; a score near 1 indicates a higher affective than cognitive ToM performance level; a score near −1 a higher cognitive than affective ToM performance level.

*Yoni-48 normative data extraction*: Mean, standard deviation, median, 25th–75th percentile, the minimum, and maximum values were computed for each composite score (ACC, RT, CA_A_, and CA_RT_).

## 3. Results

### 3.1. Participants’ demographics

In total, 235 subjects took part in the research (127 females, *χ*^2^ = 1.54, *p* = 0.215). The mean age was 41.46 ± 18.62, and the mean years of education were 15.29 ± 3.40. Table 1 reports the frequency of subjects per sex, age, and years of education groups.

**Table 1:** Subjects’ numerousness in age, sex, and education groups.

	Years of education groups					
	≤8		13		≥16	
	Males	Females	Males	Females	Males	Females
Age groups						
18–29	2	3	12	20	26	26
30–39	0	0	6	7	12	12
40–49	0	3	3	3	8	8
50–59	3	3	6	7	7	8
60–69	2	4	6	5	3	7
≥70	3	3	8	6	1	2
	10	16	41	48	57	63

### 3.2. Yoni-48 validity

#### 3.2.1. Convergent validity

Spearman ϱ correlation showed a significant association between ET and Yoni-48 total score and subscores (Yoni second-order, affective, cognitive, second-order affective, and second-order cognitive). No correlation was found between GT and Yoni-48 scores confirming the divergent validity of the tool (see Table 2 for details).

**Table 2:** Correlations between Yoni-48 scores and ET and GT.

	Yoni total	First-order	Second-order	Affective	Cognitive	First-order affective	Second-order affective	First-order cognitive	Second-order cognitive
ET ϱ (*p*)	0.292 (<0.001)	0.141 (0.033)	0.290 (<0.001)	0.289 (<0.001)	0.254 (<0.001)	0.113 (0.086)	0.289 (<0.001)	0.175 (0.008)	0.254 (<0.001)
GT ϱ (*p*)	0.004 (0.947)	−0.007 (0.915)	0.012 (0.858)	0.040 (0.543)	−0.014 (0.827)	0.012 (0.853)	0.037 (0.574)	−0.014 (0.837)	0.002 (0.975)

ϱ = Spearman’s rho. A p-value threshold of 0.005 was considered for Bonferroni’s multiple comparison correction.

#### 3.2.2. Item discrimination ability

The median of the Yoni-48 total raw score was 38. Based on the median, two groups were created (score ≥ 38: *n* = 126; score < 38: *n* = 109). The mean item discrimination ability had a moderate effect size *h* of 0.75 ± 0.24. For the first-order items, the mean *h* was moderate, 0.54 ± 0.08, while for the second-order items was high, 0.87 ± 0.21. Details are reported in Table 3.

**Table 3:** Yoni-48’s items discrimination ability.

Items	ToM component	ToM level	Accuracy	*h*
1	Affective	First-order	0.90	0.50
2	Cognitive	First-order	0.91	0.50
5	Cognitive	First-order	0.95	0.44
6	Affective	First-order	0.96	0.58
7	Cognitive	First-order	0.95	0.65
8	Affective	First-order	0.94	0.50
9	Affective	First-order	0.94	0.73
11	Affective	First-order	0.96	0.55
12	Cognitive	First-order	0.97	0.51
13	Cognitive	First-order	0.96	0.55
14	Affective	First-order	0.92	0.51
15	Affective	Second-order	0.77	0.62
16	Affective	First-order	0.93	0.48
17	Affective	First-order	0.85	0.62
18	Affective	Second-order	0.79	0.97
19	Affective	Second-order	0.85	0.89
20	Cognitive	First-order	0.85	0.55
21	Affective	Second-order	0.89	0.78
22	Affective	Second-order	0.87	0.71
23	Affective	Second-order	0.81	0.36
24	Affective	Second-order	0.69	0.70
25	Affective	Second-order	0.89	0.99
26	Cognitive	First-order	0.95	0.61
27	Cognitive	First-order	0.95	0.44
29	Affective	Second-order	0.54	0.69
30	Cognitive	Second-order	0.85	0.60
31	Cognitive	Second-order	0.91	0.69
32	Cognitive	Second-order	0.56	0.97
33	Cognitive	Second-order	0.73	1.10
34	Cognitive	Second-order	0.72	1.21
35	Affective	Second-order	0.75	1.07
36	Cognitive	Second-order	0.93	0.78
37	Affective	Second-order	0.80	1.00
38	Affective	Second-order	0.85	0.54
40	Cognitive	Second-order	0.85	1.00
41	Cognitive	Second-order	0.88	1.02
42	Cognitive	Second-order	0.88	0.86
43	Cognitive	Second-order	0.85	0.97
44	Affective	Second-order	0.82	1.10
46	Cognitive	Second-order	0.69	1.04
47	Cognitive	Second-order	0.65	1.19
48	Cognitive	Second-order	0.87	0.81

The dichotomous Rasch model showed a Pearson reliability *r* = 0.735. The mean information-weighted mean square statistic was 0.99 ± 0.12, and the outlier-sensitive means square statistic was 0.86 ± 0.37. Second-order items’ ability was acceptable (second-order items: mean infit = 1.03 ± 0.12, mean outfit = 0.95 ± 0.31), while the first-order items’ ability was low (mean infit = 0.93 ± 0.09, mean outfit = 0.71 ± 0.44).

### 3.3. Yoni-48 reliability

Internal consistency of Yoni-48 was high, considering all mental items together and Yoni sub-scales. Also, Spearman-Brown ϱ_SP_ showed high split-half reliability, adequate for utilizing the tool in a clinical context (see Table 4).

**Table 4:** Yoni-48’s inter-item and split-half reliability.

	McDonald’s *ω*	Cronbach’s *α*	Guttman’s *λ*^2^	Guttman’s *λ*^6^	Split-half Spearman-Brown ϱ_SP_	
					Median	95% HDI
Yoni total score	0.86	0.90	0.91	0.95	0.90	0.85–0.93
first-order ToM score	0.89	0.89	0.90	0.94	0.90	0.84–0.93
Second-order ToM score	0.88	0.88	0.88	0.91	0.88	0.82–0.91
Affective ToM score	0.80	0.81	0.82	0.87	0.81	0.72–0.87
Cognitive ToM score	0.80	0.84	0.86	0.90	0.85	0.76–0.90

### 3.4. Yoni-48 standardization

The multiple regression model reported a significant predictive effect of age and education on the second-order, affective, and cognitive Yoni accuracy score, while no effect of these variables was registered on the first-order accuracy score. Age was the only significant predictor of all Yoni-48 response time scores (Table 5).

**Table 5:** Demographical predictors of Yoni-48’s performance.

	Predictors	*β*	SE	95% CI lower, upper	*t*	Targeted *p*	Omnibus *p*	*R* ^2^
*Accuracy*								
First-order	Sex	−0.213	0.303	−0.81, 0.38	−0.70	0.482	0.459	0.011
	Age	−0.007	0.008	−0.02, 0.01	−0.79	0.433		
	Education (y)	0.048	0.050	−0.05, 0.15	0.96	0.336		
	Intercept	14.705	0.935	12.86, 16.55	15.72	<0.001		
Second-order	Sex	−0.773	0.610	−1.97, 0.43	−1.27	0.207	<0.001	0.141
	Age	−0.059	0.017	−0.09, −0.03	−3.48	<0.001		
	Education (y)	0.380	0.101	0.18, 0.58	3.76	<0.001		
	Intercept	17.870	1.882	14.16, 21.58	9.49	<0.001		
Affective	Sex	−0.469	0.420	−1.30, 0.36	−1.12	0.264	<0.001	0.091
	Age	−0.034	0.012	−0.06, −0.01	−2.87	0.005		
	Education (y)	0.190	0.069	0.05, 0.33	2.74	0.007		
	Intercept	16.55	1.290	14.00, 19.10	12.78	<0.001		
Cognitive	Sex	−0.517	0.422	−1.35, 0.31	−1.22	0.222	<0.001	0.108
	Age	−0.032	0.012	−0.05, −0.01	−2.74	0.007		
	Education (y)	0.238	0.070	0.10, 0.38	3.42	<0.001		
	Intercept	16.03	1.301	13.47, 18.59	12.32	<0.001		
*Response time*								
First-order	Sex	0.447	0.288	−0.12, 1.01	1.55	0.121	<0.001	0.204
	Age	0.055	0.008	0.04, 0.07	6.90	<0.001		
	Education (y)	−0.052	0.044	−0.14, 0.03	−1.18	0.239		
	Intercept	2.638	0.856	0.95, 4.32	3.08	0.002		
Second-order	Sex	0.324	0.425	−0.51, 1.16	0.76	0.447	<0.001	0.312
	Age	0.113	0.012	0.09, 0.14	9.53	<0.001		
	Education (y)	−0.070	0.065	−0.20, 0.06	−1.07	0.284		
	Intercept	5.482	1.265	2.99, 7.97	4.33	<0.001		
Affective	Sex	−8.734	7.78	−24.06, 6.59	−1.12	0.263	<0.001	0.293
	Age	1.947	0.217	1.52, 2.37	8.97	<0.001		
	Education (y)	−1.47	1.287	−4.00, 1.07	−1.14	0.256		
	Intercept	101.47	23.993	54.20, 148.74	4.23	<0.001		
Cognitive	Sex	0.308	0.349	−0.38, 0.99	0.88	0.378	<0.001	0.298
	Age	0.088	0.010	0.07, 0.11	9.05	<0.001		
	Education (y)	−0.078	0.053	−0.18, 0.03	−1.47	0.143		
	Intercept	4.766	1.037	−2.72, 6.81	4.59	<0.001		

S.E. = standard error; y = years.

### 3.5. Yoni-48 normative data extraction

Adjustment values to compute Yoni-48 adjusted scores are reported in Table 6. Formulas to adjust raw data and a calculator to automatically adjust raw data are reported in Supplementary materials S2, S3. Table 7 shows the normative data of the adjusted Yoni-48 scores.

**Table 6:** Adjustment for sex, age, and years of education of Yoni-48 raw scores.

Age	Education					
	≤8		13		16	
	M	F	M	F	M	F
*Accuracy*						
First-order						
18–29	0.32	0.11	0.08	−0.13	−0.06	−0.27
30–39	0.40	0.19	0.16	−0.05	0.01	−0.20
40–49	0.47	0.26	0.23	0.02	0.09	−0.13
50–59	0.54	0.33	0.30	0.09	0.15	−0.06
60–69	0.61	0.40	0.37	0.16	0.22	0.01
≥70	0.68	0.47	0.44	0.23	0.29	0.08
Second-order						
18–29	2.06	1.29	0.16	−0.61	−0.98	−1.75
30–39	2.71	1.94	0.81	0.04	−0.33	−1.10
40–49	3.30	2.53	1.40	0.63	0.26	−0.51
50–59	3.89	3.12	2.00	1.22	0.85	0.08
60–69	4.48	3.71	2.58	1.81	1.44	0.67
≥70	5.07	4.30	3.17	2.40	2.03	1.26
Affective						
18–29	0.99	0.52	0.04	−0.43	−0.53	−1.00
30–39	1.36	0.89	0.41	−0.06	−0.16	−0.63
40–49	1.70	1.23	0.75	0.28	0.18	−0.29
50–59	2.04	1.57	1.09	0.62	0.52	0.05
60–69	2.38	1.91	1.43	0.96	0.86	0.39
≥70	2.72	2.25	1.77	1.30	1.20	0.73
Cognitive						
18–29	1.40	0.88	0.21	−0.31	−0.51	−1.02
30–39	1.75	1.23	0.56	0.04	−0.16	−0.67
40–49	2.07	1.55	0.88	0.36	0.16	−0.35
50–59	2.39	1.87	1.20	0.68	0.48	−0.03
60–69	2.71	2.19	1.52	1.00	0.80	0.29
≥70	3.03	2.51	1.84	1.32	1.12	0.61
*Response time*						
First-order						
18–29	0.40	0.85	0.66	1.11	0.82	1.27
30–39	−0.20	0.24	0.06	0.51	0.21	0.66
40–49	−0.75	−0.30	−0.49	−0.04	−0.33	0.11
50–59	−1.30	−0.85	−1.04	−0.59	−0.88	−0.44
60–69	−1.85	−1.40	−1.59	−1.14	−1.43	−0.99
≥70	−2.40	−1.95	−2.14	−1.69	−1.98	−1.54
Second-order						
18–29	1.37	1.69	1.72	2.04	1.93	2.25
30–39	0.13	0.45	0.478	0.80	0.69	1.01
40–49	−1.00	−0.68	−0.65	−0.33	−0.44	−0.12
50–59	−2.13	−1.81	−1.78	−1.46	−1.57	−1.25
60–69	−3.26	−2.94	−2.91	−2.59	−2.70	−2.38
≥70	−4.39	−4.07	−4.04	−3.72	−3.83	−3.51
Affective						
18–29	1.15	1.58	1.38	1.81	1.52	1.96
30–39	0.11	0.55	0.35	0.78	0.49	0.92
40–49	−0.83	−0.39	−0.59	−0.16	−0.45	−0.02
50–59	−1.77	−1.33	−1.53	−1.10	−1.39	−0.96
60–69	−2.71	−2.27	−2.47	−2.04	−2.33	−1.90
≥70	−3.65	−3.21	−3.41	−2.98	−3.27	−2.84
Cognitive						
18–29	0.87	1.18	1.26	1.57	1.49	1.80
30–39	−0.10	0.21	0.29	0.60	0.53	0.83
40–49	−0.98	−0.67	−0.59	−0.28	−0.35	−0.04
50–59	−1.86	−1.55	−1.47	−1.16	−1.23	−0.92
60–69	−2.74	−2.43	−2.35	−2.04	−2.11	−1.80
≥70	−3.62	−3.31	−3.23	−2.92	−2.99	−2.68

M = males; F = females. The upper and lower limit accuracy scores have not to be adjusted.

**Table 7:** Normative data of Yoni-48 item in the Italian population.

	Accuracy	Response time (s)
*First-order score*		
Range score (minimum, maximum)	0, 16	0, 60
Mean, SD	14.98, 2.26	4.41, 2.23
Median	15.83	3.93
25th, 75th percentile	15.20, 16.00	3.12, 5.19
*Second-order score*		
Range score (minimum, maximum)	0, 26	0, 60
Mean, SD	20.68, 4.52	9.29, 3.24
Median	21.75	8.69
25th, 75th percentile	17.84, 24.11	7.16, 10.81
*Affective score*		
Range score (minimum, maximum)	0, 21	0, 60
Mean, SD	17.75, 3.13	7.46, 2.84
Median	18.72	6.99
25th, 75th percentile	16.28, 20.06	5.63, 8.98
*Cognitive score*		
Range score (minimum, maximum)	0, 21	0, 60
Mean, SD	17.92, 3.07	7.40, 2.66
Median	18.89	6.98
25th, 75th percentile	16.49, 20.26	5.70, 8.44
*Total score*		
Range score (minimum, maximum)	0, 1	0, 1
Mean, SD	0.85, 0.14	0.92,0.04
Median	0.88	0.92
25th, 75th percentile	0.78, 0.95	0.90, 0.94
*CA score*		
Range score (minimum, maximum)	−1, 1	−1, 1
Mean, SD	−0.01, 0.08	0.25, 0.09
Median	−0.01	0.23
25th, 75th percentile	−0.04, 0.03	0.19, 0.29

## 4. Discussion

The present study aimed to investigate the psychometric properties of the Italian short version (48-item) of the Yoni task (Rossetto et al., 2018). Specifically, we tested the validity, item discrimination ability, and inter-item reliability of the Yoni-48. Moreover, we provided normative data for the Italian population to enhance the reliable application of the tool in the clinical context.

Globally, the results supported the Yoni-48 validity and reliability. A high convergent validity with an established ToM test (Vellante et al., 2013) was confirmed, in line with our previous study on the Yoni-98 (Isernia et al., 2022). In detail, affective, cognitive, and second-order ToM Yoni subscales presented a highly significant association with ET, while first-order ToM subscales showed a weaker correlation. The complexity of the states of mind depicted in the ET stimuli, as well as the language demands related to the performance, renders ET an advanced ToM task (Black and An, 2019). It is plausible to assume that Yoni’s first-level ToM scores do not fully cover the wide mentalizing skills involved in a more advanced task, such as ET. Another piece of evidence supporting the validity of Yoni-48 was the absence of association with GT that presents the same stimuli of ET but requires visual perception instead of social cognition abilities. Beside the convergent validity, the item discrimination ability was extracted, confirming Yoni-48 items’ good capacity to discriminate between high and low ToM performance, especially by the second-order items. Although first-level items were not optimal in discriminating between high and low performance, including these items in the items pool would be preferable in light of Yoni’s application in the clinical context. In fact, some atypical conditions could show an impairment also in first-order ToM, and the test’s floor effects may be prevented. On the contrary, a ceiling effect in first-order ToM is expected in healthy adults (Flavell, 1999; Hughes and Leekam, 2004). Concerning the inter-item reliability, results supported a high internal consistency, also adequate for using the Yoni-48 task in the clinical context, as previously shown for Yoni-98 (Isernia et al., 2022). This was also confirmed by the split-half reliability results. One of the critical issues of ToM tasks is the absence or weak evidence supporting their psychometric proprieties, such as internal consistency. Especially a recent review (Yeh et al., 2021) highlighted a low-to-acceptable internal consistency level in 34 ToM tests, with an acceptable alpha/omega value only for three tools, the ET, faux pas, and visual jokes. In these terms, the Yoni-48 task stands out from other ToM tools for stability and reproducibility. Future studies need to further explore the reproducibility over time of the Yoni-48 with a test-retest design.

When testing the effect of demographical variables, age and years of education, but not sex, predicted the Yoni accuracy (ACC) score for both cognitive and affective second-order items but not for first-order items. Thus, higher ToM performance was associated with younger age and higher education, except for first-level ToM. This is in line with previous studies showing that the age-related decline in both cognitive and affective ToM starts from the advanced ToM tasks (for a review, see Henry et al., 2013) and that the educational level may explain differences in ToM performance (Li et al., 2013). As expected, age was the only significant predictor of Yoni-48 response time scores, which increase with age. This result can be explained by the physiological slowing of psychomotor speed commonly observed in older people (Salthouse, 2009). The Yoni-48 indexes may be adopted in future contributions investigating the link between cognitive functions and mentalizing both in typical and atypical conditions, which remains an open debate in the literature (Wade et al., 2018; Rossetto et al., 2022). In fact, while a line of evidence supports the existence of a separate cluster of ToM and executive functions processes, which are independent of each other, recent findings suggest a mixed cluster including both ToM and executive functions abilities in addition to separate clusters previously highlighted (Torralva et al., 2015; Bertoux et al., 2016; Roca, 2016). The Yoni-48 indexes would allow further exploring of the relationship between ToM and executive functions focusing on different ToM subdimensions, also taking into account of the inverse trend due to age-related differences and education influence.

To date, only a few studies provided normative data on ToM measures (see, for example, Dodich et al., 2015; Baksh et al., 2018; Delgado-Álvarez et al., 2021), and the adoption of standardized ToM measures in a clinical context remains marginal. Moreover, integrating social cognition tests in neuropsychological batteries is challenging due to the lengthy administration. Yoni-48 offers a quick and reliable ToM assessment, thanks to the digital administration modality, allowing evaluation of both ToM accuracy, response speed, and an eventual dissociation between affective and cognitive components. Our normative data suggested a high accuracy level, response speed, and a high balance between affective and cognitive components of ToM in the healthy Italian population. In addition, future studies need to investigate the tool characteristics in clinical populations. In fact, in the clinical context, adopting the Yoni-48 for detecting specific deficits in separate components of ToM and for the implementation of tailored rehabilitation activities may be useful (Henry et al., 2016).

This study is not without limitations. Our participants’ group did not include younger people with very low education, such as subjects aged 30–39 with less than 8 years of education. However, it has to be mentioned that middle school in Italy consists of compulsory education, and young adults without middle school education are under-represented. Also, although this study supported the psychometric proprieties of Yoni-48 also in the clinical context, additional studies are essential to confirm the sensitivity and specificity of this tool in detecting mentalizing deficits also in clinical samples (diagnostic validity). Although further work is needed before adopting Yoni-48 for clinical populations, its digital administration modality aligns it with the new digital assessment perspective in neuropsychology (Bilder, 2011; American Psychiatric Association, 2013).

In conclusion, this study supports the psychometric properties of the Yoni-48 in terms of validity and reliability and provides normative data for the Italian population. Further studies are needed to test the suitability of this short version for profiling the social cognition neurocognitive phenotype and exploring the performance level of Yoni-48 in the clinical population.

## Data availability statement

The raw data supporting the conclusions of this article will be made available by the authors, without undue reservation.

## Ethics statement

The studies involving human participants were reviewed and approved by Don Gnocchi Foundation Ethics Committee. The patients/participants provided their written informed consent to participate in this study.

## Author contributions

FB, FR, and SI conceived the study. FR and SI collected data and carried out the study and wrote the draft of the manuscript. SI performed the statistical analysis. AM, FB, and SS-T substantively revised and edited the draft of the manuscript. All authors read and approved the final version of the manuscript.

## Funding

This work was supported by 5x1000 funds – 2020, Italian Ministry of Health – Ricerca Corrente.

## Conflict of interest

The authors declare that the research was conducted in the absence of any commercial or financial relationships that could be construed as a potential conflict of interest.

## Publisher’s note

All claims expressed in this article are solely those of the authors and do not necessarily represent those of their affiliated organizations, or those of the publisher, the editors and the reviewers. Any product that may be evaluated in this article, or claim that may be made by its manufacturer, is not guaranteed or endorsed by the publisher.
