# Supplementary material for: Standardization and normative data of the 48-item Yoni short version for the assessment of theory of mind in typical and atypical conditions
Source: Front Aging Neurosci. 2023 Jan 12;14:1048599. doi: 10.3389/fnagi.2022.1048599 (PMC9877508; doi:10.3389/fnagi.2022.1048599)
Supplement: Supplementary file 1 [file Data_Sheet_1.docx]

Supplementary Material

“Standardization and Normative Data of the 48-items Yoni short version for the Assessment of Theory of Mind in Typical and Atypical conditions”

# S1. Instruction to score Yoni-48

*
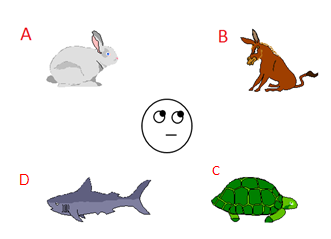
*

| Yoni-48  item | ToM  Component | Level of attribution | Scoring |
| --- | --- | --- | --- |
| **1** | AFF | 1ORD | A |
| **2** | COG | 1ORD | B |
| **3** | PHY | 1ORD | B |
| **4** | PHY | 1ORD | C |
| **5** | COG | 1ORD | C |
| **6** | AFF | 1ORD | D |
| **7** | COG | 1ORD | D |
| **8** | AFF | 1ORD | A |
| **9** | AFF | 1ORD | B |
| **10** | PHY | 1ORD | D |
| **11** | AFF | 1ORD | D |
| **12** | COG | 1ORD | D |
| **13** | COG | 1ORD | A |
| **14** | AFF | 1ORD | B |
| **15** | AFF | 2ORD | C |
| **16** | AFF | 1ORD | D |
| **17** | AFF | 1ORD | D |
| **18** | AFF | 2ORD | B |
| **19** | AFF | 2ORD | B |
| **20** | COG | 1ORD | C |
| **21** | AFF | 2ORD | A |
| **22** | AFF | 2ORD | D |
| **23** | AFF | 2ORD | A |
| **24** | AFF | 2ORD | D |
| **25** | AFF | 2ORD | D |
| **26** | COG | 1ORD | D |
| **27** | COG | 1ORD | B |
| **28** | PHY | 2ORD | C |
| **29** | AFF | 2ORD | A |
| **30** | COG | 2ORD | D |
| **31** | COG | 2ORD | A |
| **32** | COG | 2ORD | A |
| **33** | COG | 2ORD | B |
| **34** | COG | 2ORD | A |
| **35** | AFF | 2ORD | B |
| **36** | COG | 2ORD | A |
| **37** | AFF | 2ORD | B |
| **38** | AFF | 2ORD | A |
| **39** | PHY | 2ORD | D |
| **40** | COG | 2ORD | B |
| **41** | COG | 2ORD | C |
| **42** | COG | 2ORD | C |
| **43** | COG | 2ORD | D |
| **44** | AFF | 2ORD | C |
| **45** | PHY | 2ORD | A |
| **46** | COG | 2ORD | B |
| **47** | COG | 2ORD | D |
| **48** | COG | 2ORD | C |
|  |  |  |  |

*Yoni-48 = 48-items short version of Yoni. AFF = Affective ToM; COG = Cognitive ToM; 1ORD = First-Order ToM; 2ORD = Second-Order ToM.*

# S2. Formula to compute adjusted scores of Yoni-48

***Accuracy scores***

Yoni First Order adjusted score:

Males: x- [(age-41.46)* -0.007] – [(education – 15.28)* 0.048] + [(0-0.54) *-0.213)

Females: x- [(age-41.46)* -0.007] – [(education – 15.28)* 0.048] + [(1-0.54) *-0.213)

Yoni Second Order adjusted score:

Males: x- [(age-41.46)* -0.059] – [(education – 15.28)* 0.38] + [(0-0.54)*- 0.773]

Females: x- [(age-41.46)* -0.059] – [(education – 15.28)* 0.38] + [(1-0.54)*- 0.773]

Yoni Affective adjusted score:

Males: x- [(age-41.46)* -0.034] – [(education – 15.28)* 0.19] + [(0-0.54)*- 0.469]

Females: x- [(age-41.46)* -0.034] – [(education – 15.28)* 0.19] + [(1-0.54)*-0.469]

Yoni Cognitive adjusted score:

Males: x- [(age-41.46)* -0.032] – [(education – 15.28)* 0.238] + [(0-0.54)*- 0.517]

Females: x- [(age-41.46)* -0.032] – [(education – 15.28)* 0.238] + [(1-0.54)*-0.517]

***Response Time scores***

Yoni First Order adjusted score:

Males: x- [(age-41.46)* 0.055] – [(education – 15.28)* -0.052] + [(0-0.54) *0.447]

Females: x- [(age-41.46)* 0.055] – [(education – 15.28)* -0.052] + [(1-0.54) *0.447]

Yoni Second Order adjusted score:

Males: x- [(age-41.46)* 0.113] – [(education – 15.28)* -0.070] + [(0-0.54) *0.324]

Females: x- [(age-41.46)* 0.113] – [(education – 15.28)* -0.070] + [(1-0.54) *0.324]

Yoni Affective adjusted score:

Males: x- [(age-41.46)* 0.094] – [(education – 15.28)* -0.047] + [(0-0.54) *0.433]

Females: x- [(age-41.46)* 0.094] – [(education – 15.28)* -0.047] + [(1-0.54) *0.433]

Yoni Cognitive adjusted score:

Males: x- [(age-41.46)* 0.088] – [(education – 15.28)* -0.078] + [(0-0.54) *0.308]

Females: x- [(age-41.46)* 0.088] – [(education – 15.28)* -0.078] + [(1-0.54) *0.308]
